# Supplementary material for: BOAS in the Boston Terrier: A healthier screw-tailed breed?
Source: PLoS One. 2024 Dec 31;19(12):e0315411. doi: 10.1371/journal.pone.0315411 (PMC11687697; doi:10.1371/journal.pone.0315411)
Supplement: S1 Appendix — (DOCX) [file pone.0315411.s003.docx]

**Repeatability Study Methodology**

To test the repeatability of the conformational measurements, a study was undertaken to assess agreement of repeated measures. This was performed in two ways. Firstly, to assess repeatability of the photographic measurements by the same observer (FT), as these measurements were to be used for analysis into BOAS risk factors. Secondly, a study was also undertaken to assess the agreement between different investigators (FT, JL & DS) in order to assess the replicability of results under different observers to look into whether these conformational measurements may be used as tools to select which dogs are more likely to be affected by BOAS. Measurements were repeated by three observers for the photographic measurements and by two observers for soft tape measurements. A methods comparison study was also performed for the CFR measurements as this study describes the use of a novel way to measure CFR using photographs to test the validity of its use.

Dogs included in this study were taken from a wider database and included a number of different breeds. For the inter-rater repeatability of the soft tape measurements, thirty dogs (n=30) were selected that had been available for repeated measurements at time of assessment. For the intra- and inter-rater repeatability studies of photographic measurements, a random number generator was used to select the subject record study ID numbers. A total of 20 dogs were selected (Griffon Bruxellois n=2, Chihuahua n=2, Affenpinscher n=3, King Charles Spaniel n=4, Shih Tzu n=3, Cavalier King Charles Spaniel n=1, Japanese Chin n=1, 1 Boxer n=1, 1 Boston Terrier n=1, Maltese n=1, Pomeranian n=1). For the method comparison study, a total of forty-one (n=41) dogs were compared (Affenpinscher n=3, Boston Terrier n=2, Boxer n=5, Chihuahua n=3, Cavalier King Charles Spaniel n=5, Griffon Bruxellois n=3, Japanese Chin n=3, King Charles Spaniel n=5, Maltese n=2, Pekingese n=1, Pomeranian n=4, Shih Tzu n=5). Due to poor repeatability of skull width for the skull index calculation, dorsal plane measurement inter-rater repeatability was repeated with smooth coated breeds only. It was observed that in the more coarsely coated dogs the occiput and stop were difficult to define from the dorsal perspective. This smaller study used a total of eight dogs from three breeds (Boxer n=2, Boston Terrier n=3, Staffordshire Bull Terrier n=3).

Intra-class correlation coefficient was calculated to test the agreement of the measurements taken for the same subject for analysis of intra-rater repeatability (FT) and inter-rater repeatability of three different raters (FT, JL & DS)(58). (The R function code can be found via <https://rdrr.io/cran/irr/src/R/icc.R>. The following criteria was used for interpretation of ICC value (58).

< 0.5: poor reliability
0.5 - 0.75: moderate reliability
0.75 - 0.9: good reliability
> 0.90: excellent reliability

Results for intra-class correlation coefficient shown in S1 Table.

For the methods comparison study, equivalent measurements were taken from computed tomography (CT) scans that were available for dogs participating in the study. The CT images taken from each subject were aligned under multiplanar reconstruction to ensure precise alignment to the planes. The muzzle length and cranial length were also measured on the midline sagittal image. Muzzle length was identified as the linear distance from the edge of the rostrum to the stop. CT and photographic measurements were plotted on a graph correlation analysis performed to check for significance. Bland-Altman method comparison analysis was used to assess the difference between photographic versus computed tomographic measurements and compare to the average. The bias of the photographic measurements and standard deviation of the bias were calculated. Results shown in S1 Figure.
