# Supplementary material for: BOAS in the Boston Terrier: A healthier screw-tailed breed?
Source: PLoS One. 2024 Dec 31;19(12):e0315411. doi: 10.1371/journal.pone.0315411 (PMC11687697; doi:10.1371/journal.pone.0315411)
Supplement: S1 Table — (A) Intra-rater repeatability results for one rater across up to 19 subjects of multiple dog breeds in the photographic measurements. (B) Inter-rater repeatability results for one rater across up to 20 subjects of multiple dog breeds in the photographic measurements. Skull index [1] displaying ICC result for analysis performed on multiple dog breeds, and skull index [2] displaying ICC value for analysis performed on smooth-coated breeds (Boston Terrier, Boxer and Staffordshire Bull Terrier) only. (C) Inter-rater repeatability results for soft tape measurements conducted by two raters across 30 subjects of multiple dog breeds. (DOCX) [file pone.0315411.s004.docx]

| **Measurement** | 1. **Intra-RR** | | | | |
| --- | --- | --- | --- | --- | --- |
|  | ICC | CI (95%) | F | Sample size | p value |
| Skull width (fp) | 0.984 | 0.967 - 0.993 | 18,37.6 | 19 | 6.34E-23 |
| Intercanthal distance | 0.986 | 0.968 - 0.995 | 15,30.2 | 16 | 5.20E-19 |
| Craniofacial ratio | 0.992 | 0.982 - 0.996 | 18,36.2 | 19 | 2.31E-26 |
| Skull index | 0.948 | 0.879 - 0.981 | 14,29.6 | 15 | 2.53E-11 |
|  | 1. **Inter-RR (3 measurement average)** | | | | |
| **Measurement** | ICC | CI (95%) | F | Sample size | p value |
| Skull width (fp) | 0.732 | 0.399 - 0.892 | 17,10.3 | 18 | 4.79E-05 |
| Intercanthal distance | 0.977 | 0.95 - 0.991 | 16,33.9 | 17 | 1.05E-25 |
| Craniofacial ratio | 0.823 | 0.505 - 0.934 | 19,7.95 | 20 | 2.84E-05 |
| Skull index [1] | 0.381 | 0.067 - 0.708 | 12,24 | 13 | 0.00746 |
| Skull index [2] | 0.852 | 0.609 – 0.965 | 7, 15.4 | 8 | 1.24e-06 |
|  | 1. **Inter-RR** | | | | |
| **Measurement** | ICC | CI (95%) | F | Sample size | p value |
| Body length | 0.908 | 0.817 – 0.955 | 21.4,29.3 | 30 | 4.83e-13 |
| Body height | 0.979 | 0.956 – 0.990 | 91.2,29.5 | 30 | 5.48e-22 |
| Neck girth | 0.769 | 0.573 – 0.883 | 7.65,30 | 30 | 1.54e-07 |
| Chest girth | 0.935 | 0.870 – 0.968 | 29.7,30 | 30 | 2.94e-15 |
| Tail length | 0.918 | 0.830 – 0.961 | 25.4,25.4 | 30 | 1.31e-12 |
