# Supplementary material for: BOAS in the Boston Terrier: A healthier screw-tailed breed?
Source: PLoS One. 2024 Dec 31;19(12):e0315411. doi: 10.1371/journal.pone.0315411 (PMC11687697; doi:10.1371/journal.pone.0315411)
Supplement: S2 Table — Mixed-effects model (REML) and unpaired two-tailed t-tests comparing the differences in change in MEF rate between each sample group. Paired two tailed t-tests comparing MEF rates across different time periods of the expiratory breath within each sample group. (DOCX) [file pone.0315411.s005.docx]

|  |  | **Group Comparison** | **Controls vs. Grade 0s** | **Controls vs. Grade 1-3s** | **Grade 0s vs. Grade 1-3s** |
| --- | --- | --- | --- | --- | --- |
| **Mixed-effects model (REML)** | **Fixed Effects: P value** | **Time** | 0.008** | 0.139 | 0.305 |
|  |  | **Group** | 0.167 | 0.052 | 0.413 |
|  |  | **Time x Group** | 0.385 | 0.008** | 0.0006*** |
|  | **Random Effects: Std. Dev.** | **Subject** | 2.99 | 3.62 | 3.62 |
|  |  | **Residual** | 1.45 | 2.83 | 2.83 |
|  |  |  |  |  |  |
| **Unpaired two tailed t-test** | **Change in flow rate: raw P values** | **0.25-0.75** | 0.402 | 0.015* | 0.003** |
|  |  | **0.25-0.5** | 0.071 | 0.059 | 0.007** |
|  |  | **0.5-0.75** | 0.83 | 0.095 | 0.208 |
|  | **Change in flow rate: adjusted P values** | **0.25-0.75** | 0.643 | 0.043* | 0.010* |
|  |  | **0.25-0.5** | 0.198 | 0.115 | 0.014* |
|  |  | **0.5-0.75** | 0.830 | 0.115 | 0.208 |
|  |  |  |  |  |  |
|  |  |  |  |  |  |
| **Group** | **Time Period** | **Mean of Change in Flow Rate [Std. Dev]** | **95% CI of mean** | **Paired two-tailed t-test** | |
|  |  |  |  | **Raw P values** | **Adjusted P values** |
| **Control** | **0.25-0.75** | 1.08 [0.865] | 0.459 - 1.7 | 0.003** | 0.010* |
|  | **0.25-0.5** | 0.428 [0.84] | -0.173 - 1.03 | 0.142 | 0.142 |
|  | **0.5-0.75** | 0.65 [0.713] | 0.14 - 1.16 | 0.018* | 0.053 |
| **Grade 0 BTs** | **0.25-0.75** | 2 [3.33] | 0.0822 - 3.92 | 0.042* | 0.074 |
|  | **0.25-0.5** | 1.53 [1.68] | 0.557 - 2.5 | 0.005** | 0.014* |
|  | **0.5-0.75** | 0.476 [2.45] | -0.937 - 1.89 | 0.480 | 0.512 |
| **Grade 1-3 BTs** | **0.25-0.75** | -4.23 [6.21] | -8.17 - (-0.286) | 0.038* | 0.074 |
|  | **0.25-0.5** | -3.45 [6.06] | -7.31 - 0.397 | 0.074 | 0.143 |
|  | **0.5-0.75** | -0.776 [2.48] | -2.35 - 0.798 | 0.301 | 0.512 |
