# Supplementary material for: BOAS in the Boston Terrier: A healthier screw-tailed breed?
Source: PLoS One. 2024 Dec 31;19(12):e0315411. doi: 10.1371/journal.pone.0315411 (PMC11687697; doi:10.1371/journal.pone.0315411)
Supplement: S3 Table — BOAS Grade 0 Boston terriers and BOAS Grade 1–3 Boston Terriers. (DOCX) [file pone.0315411.s006.docx]

| **Variable** | **BOAS Grade** | **Median [min – max]** | **Mean [SD]** | **95% CI** |
| --- | --- | --- | --- | --- |
| **Body length (cm)** | 0 | 32.0 [25.0 – 43.0] | 31.9 [3.72] | 30.7 – 33.1 |
|  | 1-3 | 31.5 [25.0 – 43.0] | 31.7 [3.70] | 30.8 – 32.6 |
| **Body height (cm)** | 0 | 38.0 [32.0 – 45.0] | 38.2 [3.40] | 37.1 – 39.1 |
|  | 1-3 | 37.5 [30.0 – 47.0] | 37.6 [3.50] | 36.7 – 38.5 |
| **NGR** | 0 | 0.62 [0.54 – 0.68] | 0.62 [0.03] | 0.60 – 0.62 |
|  | 1-3 | 0.64 [0.52 – 0.76] | 0.63 [0.04] | 0.63 – 0.64 |
| **Tail length (cm)** | 0 | 3.0 [0.0 – 8.0] | 3.4 [2.4] | 2.62 – 4.24 |
|  | 1-3 | 2.0 [0.0 – 8.0] | 2.7 [2.0] | 2.17 – 3.21 |
| **EWR** | 0 | 0.56 [0.48 – 0.74] | 0.56 [0.05] | 0.54 – 0.58 |
|  | 1-3 | 0.55 [0.47 – 0.61] | 0.55 [0.03] | 0.54 – 0.56 |
| **SI** | 0 | 0.95 [0.81 – 1.10] | 0.96 [0.06] | 0.94 – 0.97 |
|  | 1-3 | 1.00 [0.81 – 1.22] | 1.01 [0.08] | 0.99 – 1.03 |
| **CFR** | 0 | 0.15 [0.05 – 0.22] | 0.15 [0.04] | 0.14 – 0.16 |
|  | 1-3 | 0.12 [0.05 – 0.16] | 0.12 [0.03] | 0.11 – 0.12 |
| **Age (years)** | 0 | 2.5 [1.0 – 7.0] | 3.3 [2.0] | 2.67 – 3.97 |
|  | 1-3 | 3.0 [1.0 – 10.0] | 4.0 [2.6] | 3.33 – 4.61 |
| **Weight (kg)** | 0 | 8.2 [5.1 – 11.6] | 8.2 [1.6] | 7.69 – 8.75 |
|  | 1-3 | 7.7 [4.2 – 15.8] | 8.2 [2.2] | 7.62 – 8.71 |
