# Supplementary material for: BOAS in the Boston Terrier: A healthier screw-tailed breed?
Source: PLoS One. 2024 Dec 31;19(12):e0315411. doi: 10.1371/journal.pone.0315411 (PMC11687697; doi:10.1371/journal.pone.0315411)
Supplement: S4 Table — (DOCX) [file pone.0315411.s007.docx]

| **Variable** | **Equal Variance**  **(p value of F test)** | **p-value** | **t-value** | **df** |
| --- | --- | --- | --- | --- |
| **Body length** | Yes | 0.373 | 0.324 | 100 |
| **Body height** | Yes | 0.383 | 0.876 | 100 |
| **NGR** | Yes | 0.039 * | 1.781 | 99 |
| **Tail length** | Yes | 0.052 | 1.643 | 98 |
| **EWR** | No (p=0.005) | 0.147 | 1.060 | 51.1 |
| **SI** | No (p=0.023) | 0.0002 *** | 3.689 | 92.3 |
| **CFR** | No (p=0.008) | <0.0001 **** | 4.451 | 53.3 |
| **Age** | Yes | 0.180 | 1.350 | 102 |
| **Weight** | Yes | 0.888 | 0.141 | 102 |
