# Supplementary material for: BOAS in the Boston Terrier: A healthier screw-tailed breed?
Source: PLoS One. 2024 Dec 31;19(12):e0315411. doi: 10.1371/journal.pone.0315411 (PMC11687697; doi:10.1371/journal.pone.0315411)
Supplement: S5 Table — (DOCX) [file pone.0315411.s008.docx]

| **Model** |  |  |  |  |
| --- | --- | --- | --- | --- |
| **Parameter estimates** | **Variable** | **Estimate** | **Std. Error** | **95% CI** |
| β0 | Intercept | -12.8 | 7.12 | -27.8 to 0.544 |
| β1 | Nostril Stenosis | 0.922 | 0.402 | 0.169 to 1.77 |
| β2 | Scleral Show | 1.15 | 0.633 | -0.0552 to 2.46 |
| β3 | NGR | 0.133 | 0.0836 | -0.0235 to 0.308 |
| β4 | Tail length | -0.129 | 0.129 | -0.392 to 0.121 |
| β5 | SI | 0.0713 | 0.0445 | -0.0132 to 0.165 |
| β6 | CFR | -0.278 | 0.106 | -0.506 to -0.0847 |
|  |  |  |  |  |
| **Odds ratios** | **Variable** | **Estimate** | **95% CI** |  |
| β0 | Intercept | 2.64e-006 | 8.70e-013 to 1.72 |  |
| β1 | Nostril Stenosis | 2.51 | 1.18 to 5.85 |  |
| β2 | Scleral Show | 3.17 | 0.946 to 11.7 |  |
| β3 | NGR | 1.14 | 0.977 to 1.36 |  |
| β4 | Tail length | 0.879 | 0.676 to 1.13 |  |
| β5 | SI | 1.07 | 0.987 to 1.18 |  |
| β6 | CFR | 0.757 | 0.603 to 0.919 |  |
|  |  |  |  |  |
| **Sig. diff. than zero?** | **Variable** | **\|Z\|** | **P value** |  |
| β0 | Intercept | 1.80 | 0.0713 |  |
| β1 | Nostril Stenosis | 2.29 | 0.0218* |  |
| β2 | Scleral Show | 1.82 | 0.0683 |  |
| β3 | NGR | 1.59 | 0.1121 |  |
| β4 | Tail length | 1.00 | 0.3175 |  |
| β5 | SI | 1.60 | 0.1092 |  |
| β6 | CFR | 2.63 | 0.0086** |  |
|  |  |  |  |  |
| **Model diagnostics** | | **Degrees of Freedom** | **AICc** |  |
| **Intercept-only model** | | 86 | 118 |  |
| **Selected model** | | 80 | 92.3 |  |
|  |  |  |  |  |
|  |  |  |  |  |
| **Classification table** | **Predicted 0** | **Predicted 1** | **Total** | **% Correctly classified** |
| **Observed 0** | 25 | 9 | 34 | 73.5 |
| **Observed 1** | 6 | 47 | 53 | 88.7 |
| **Total** | 31 | 56 | 87 | 82.8 |
|  |  |  |  |  |
| **Negative predictive power (%)** | | 80.6 |  |  |
| **Positive predictive power (%)** | | 83.9 |  |  |
|  |  |  |  |  |
|  |  |  |  |  |
| **Pseudo R squared** | |  |  |  |
| **Tjur's R squared** | | 0.402 |  |  |
