# Supplementary figures and images for: BOAS in the Boston Terrier: A healthier screw-tailed breed?
Source: PLoS One. 2024 Dec 31;19(12):e0315411. doi: 10.1371/journal.pone.0315411 (PMC11687697; doi:10.1371/journal.pone.0315411)

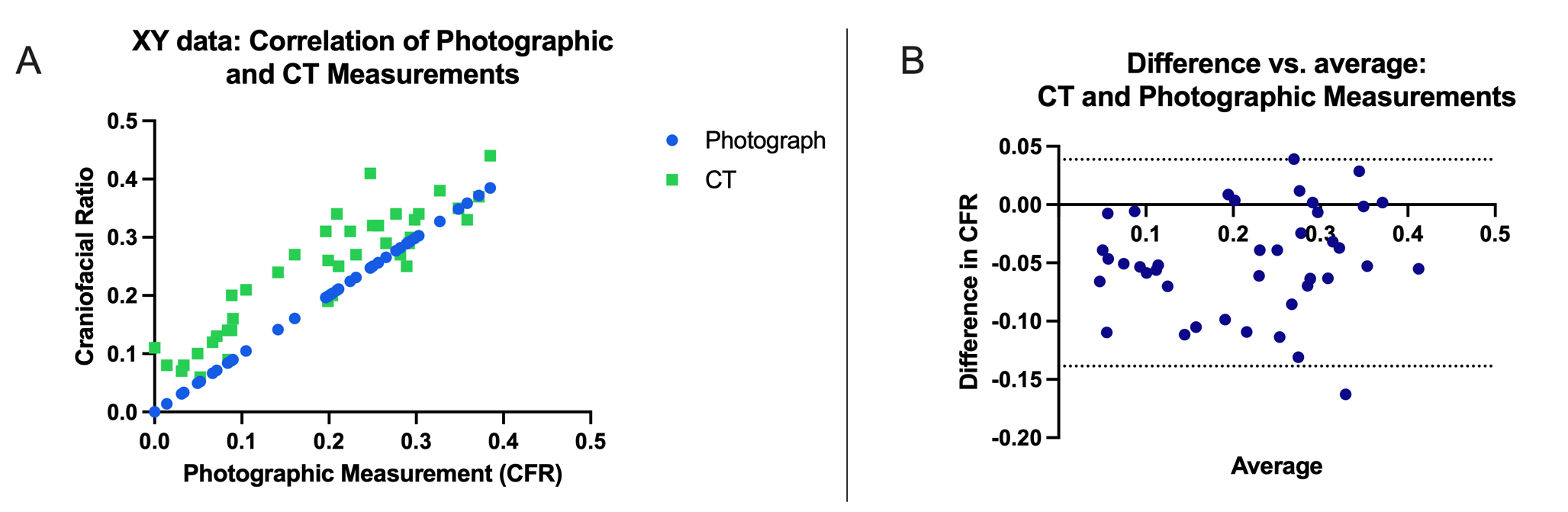

Supplement: S1 Fig — (A) Plotted craniofacial ratios of the same subjects measured through CT and photographic images. (B) Bland-Altman analysis of CT versus photographic measurements demonstrating risk of bias. CT and photographic measurements of the craniofacial ratio of the same subjects were significantly correlated (p < 0.0001). The left x axis shift of the plotted CT data indicates the underestimation bias of the photographic measurements. Bland-Altman analysis revealed a bias of -0.0499 (SD: 0.0452) for the photographic measurements when compared to the CT measurements. (TIF) [file pone.0315411.s009.tif]
