# Supplementary material for: BOAS in the Boston Terrier: A healthier screw-tailed breed?
Source: PLoS One. 2024 Dec 31;19(12):e0315411. doi: 10.1371/journal.pone.0315411 (PMC11687697; doi:10.1371/journal.pone.0315411)
Supplement: Attachment — Submitted filename: Response to Reviewers.docx [file pone.0315411.s010.docx]

PONE-D-24-10830

BOAS in the Boston Terrier: a healthier screw-tailed breed?

PLOS ONE

Dear Ioannis Savvas,

Many thanks for your response to our manuscript submission. We thank the reviewers for their comments and feedback.

The manuscript and reference list have been reviewed to ensure they meet PLOS ONE’s style requirements. We attach the revised manuscript and below we address the points raised by the reviewers.

We hope that the revised manuscript will now be suitable for publication and look forward to hearing from you.

Kind regards,

…………………..

Reviewer 1

In response to Reviewer #1’s comments, additional remarks regarding the limitations of the study and potential ways to address this have been added on lines 526-530.

Reviewer 2

The following answers are made in response to Reviewer #2’s comments organised by line originally referenced:

Line 250 - A one-sided test was chosen as it was hypothesised that Boston Terriers had a higher percentage of Grade 0 Boston Terriers (lines 97-98).

Line 256 - Amended lines 164-165 to ensure consistent language used throughout text.

Line 257 - Repeated measures ANOVA with a mixed model approach was used to look at the temporal trends between the three groups (control, Grade 0 and Grade 1-3). Using Prism, I don’t believe that would be possible with this type of analysis. The mixed effects model used accounts for the subjects being random effects to enable analysis of temporal trends, whilst each subject can start from a different flow rate level. Pairwise comparisons were therefore then used to look at the data in more granular detail. The unpaired t-tests were performed for comparing the flow rate change between groups, and paired t-tests for flow rate change within groups.

Line 261-2 - Amended sentence.

Line 272 - A one-sided test was used for continuous risk factors as it was hypothesised that more ‘extreme’ conformation was associated with BOAS, and is more sensitive at detecting a smaller effect size.

Line 273 - EWR, SI and CFR are listed on line 276.

Line 339 - Amended to ‘smaller change’ on line 340.

Lines 341-2 - Amended on line 343 to clarify that talking about the two groups (control and Grade 0).

Line 365 - Whilst it is likely difficult to differentiate on a practical level the difference between a Boston Terrier with a neck girth ratio of 0.62 compared to 0.63, noting this as a significant factor could help to identify dogs that have a more extreme thicker neck compared to chest girth as potentially being at risk of BOAS (the maximum NGR Grade 1-3 was 0.76 compared to 0.68 for Grade 0s).
